# Molar Pregnancy—An Anomalous Case

**Published:** 1879-09

**Authors:** O. E. Herrick


### MOLAR PREGNANCY—AN ANOMALOUS CASE.

By O. E. HERRICK, M.D.

On the 15th of April last I was consulted by Mrs. D. for a persistent pain in the head, and occasionally attended, after three or four hours of severe pain, by paroxysms of frenzy, which would continue for from two hours to twelve or fourteen, when they would again gradually subside. These paroxysms were always attended by a feeling of fulness about the head. She stated, upon her first visit to my office, that she was pregnant about four months, and that her headache dated from about the time of conception; she believed that her headache and spams would kill her if she was allowed to go to full time.

I thought that perhaps it was but a *ruse* to get me to produce an abortion, and felt quite sure that such was the case when she added that she had importuned her usual medical attendant (a reputable physician) to produce an abortion to relieve her, and he had refused, telling her that her headache and paroxysms of frenzy were entirely independent of her pregnancy. One thing which made me suspect, however, that the case was not a case of ordinary pregnancy, was, that she flowed a little at each monthly period, and her breasts were flaccid, although she was a woman of full habit, while she had leucorrhœa almost continuously.

I heard nothing further from her until May 2d, when I was called in haste to see her. I found her suffering severe pain in the head, face flushed, and with symptoms of mania well marked, although there were lucid intervals, but upon the return of pain she would again become violent. I gave her twenty grains of chloral hydrate every two hours, and fl. ext. of ergot, fifteen drops, every hour (I gave the last to lessen the circulation in the brain.) After the third dose of chloral she became quiet, and shortly dropped off into a sleep, and slept until about midnight quite naturally, when she awoke; and although she was still delirious, was much more quiet than before. She was given another twenty gr. dose of chloral, and in about twenty minutes again fell asleep, and slept until morning, at which time I again saw her, and found her rational, and suffering only slight pain in the head. I then made a vaginal examination, and found the uterus enlarged to about the size it should attain at a five month's pregnancy; but instead of having the hard feel of the pregnant uterus; it had a flabby, spongy feel; the os was dilated so that it readily admitted the finger, and I found that the neck was occupied by something which appeared to the finger like a piece of placenta. I introduced a gum-elastic catheter into the cavity of the uterus, and injected through it, with a common bulb syringe, about eight ounces of warm water; also continued the ergot. In about three hours I called again and found her flowing quite liberally, and undergoing pains at regular intervals. After waiting about an hour, and after a pain of unusual intensity, I made an examination, and found laying in the vagina a fleshy, fibrous mass covered externally by a membrane which might have been the decidua in a natural condition, while the inner surface of the mass was lined by a fine membrane, having the usual character of the amnion. After passing the mass, her pains suddenly stopped, and the flowing was only very moderate. I then left her, with instructions to take the ergot at longer intervals, about three hours apart. I called the next morning, and found she had passed three more, almost exactly like the first, about two inches in diameter, and she continued passing them for four days, until she had passed eleven of them, the last three of which I removed with the wire scoop; during all this time her flowing was quite moderate, and at no time severe. She made a rapid and good recovery, and has had no return of head symptoms since.

It is not an unusual thing for the uterus to be filled with a large number of vesicles of irregular shape and size, in the hydatid form of mole pregnancy. But it is, I believe, an unheard of thing for the variety described above to occur in such numbers, and each one entirely distinct from the other. It would seem as though a whole crop of ovules had been blighted, and that each one had commenced life with a distinct and separate placenta.

Another interesting feature of this case is the neurasthenia. The condition of this patient was exactly the opposite from the condition of patients said to be suffering from puerperal mania, as described by authors upon that subject. She was a robust woman, plethoric and strong, not weakened by hemorrhage or other abnormal discharges, except, perhaps, the leucorrhœa, and that had not weakened her, as her circulation was unusually strong during all her trouble. Authorities tell us that the cause of puerperal mania is anæmia, caused by hemorrhage, malignant disease of the uterus, abortion, etc., and is a disease which arises from great exhaustion. The question I wish to raise is, may it not occur from exactly the opposite condition; instead of the brain being anæmic, and not having enough blood supplied it, may it not have too much, as is the case in alcoholism, etc.? I believe both these conditions may produce maniacal excitement, and that one is likely to produce it as the other, and hence the necessity of recognizing the difference of origin before commencing treatment. Diseases of the uterus, of all kinds, seem to exert a powerful influence upon the whole nervous system, and the brain being the great nerve centre, is especially apt to be more or less influenced by any severe or protracted disease of the uterus; it need not necessarily be pregnancy, nor need it occur only after a tedious delivery; but may, and frequently does, occur from any long standing inflammation or congestion of that organ; I have seen it in bad cases of dysmenorrhœa; I have also seen it as melancholia, in cases of displacements of long standing. We frequently observe it among frail married ladies living with strong and vigorous husbands, occurring from excessive coitus; the uterus is kept in a constant state of congestion, and the woman is converted into a maniac, and hates the man she should love better than all others, and for no other reason than that her brain is influenced by her much-abused uterus. No matter what, in each individual case, the disease may be called, it is simply mania. The fact that the woman can no longer control her mind is sufficient evidence that she is insane. That point once settled in the mind of the attending physician, he should always look after the state of his patient's uterus. If he finds that his patient has recently passed through childbirth, and is suffering from puerperal fever, he should treat her for that disease. If his patient is in labor, and is attacked with violent pain, heat and throbbing of the head, which is greatly increased by her uterine pains, the cerebral excitement becoming intense, followed by furious delirium or convulsions, then he should deliver at once, and treat her for ordinary convulsions, which is but another form of this same class of difficulties. If the patient has dysmenorrhœa, and loses control of her mind, call it hysteria if you will, but it is uterine mania, just the same. Do not leave her to get well the best she may, but treat her dysmenorrhœa, and you will undoubtedly cure her mental difficulty. A patient with displaced uterus should have it replaced, and retained there after some common sense method. If the woman's husband is the cause of her trouble, either send him or her on a visit, and her peculiar form of insanity will probably vanish. In short, treat each individual case, and ignore the classes.—*Medical and Surgical Reporter.*

